# Proteomic profile of serum from patients with schizophrenia spectrum disorders

**DOI:** 10.7717/peerj.13907

**Published:** 2022-08-30

**Authors:** Elena Dmitrieva, Liudmila Smirnova, Alexander Seregin, Victor Zgoda, Arkadij Semke, Svetlana Ivanova

**Affiliations:** 1 Laboratory of Molecular Genetics and Biochemistry, Mental Health Research Institute, Tomsk National Research Medical Center of the Russian Academy of Sciences, Tomsk, Russia; 2 Laboratory of Systems Biology, Institute of Biomedical Chemistry, Moscow, Russia

**Keywords:** Schizophrenia, Schizotypal disorder, Proteomics, Mass spectrometry, System biology

## Abstract

This article describes the most likely classes of proteins and molecular processes that specifically characterize schizophrenic spectrum disorders such as simple and paranoid schizophrenia, schizotypal disorder, and acute polymorphic psychotic disorder (APPD). The identification of patients’ serum proteins was carried out using mass spectrometry. For patients with paranoid schizophrenia, the proteins responsible for translation and transcription are characteristic. A significant part of the proteins of patients with simple schizophrenia regulate the cell’s main metabolic and transport processes. These are proteins of the receptor system, vesicular transport, and extracellular matrix, which mainly carry out catabolic processes. The proteins of patients with schizotypal disorder mostly coincided with the classes of other patients, apart from chaperone proteins, which were not found in other studied groups. These proteins are mainly involved in anabolic processes. The main classes of proteins found in patients with APPD are responsible for the metabolism of nucleic acids. Active apoptosis processes were also revealed in these patients. These results from our basic knowledge about the molecular mechanisms of the pathogenesis of these disorders.

## Introduction

Schizophrenia and schizophrenia spectrum disorders are multifactorial diseases characterized by delusions, hallucinations, affective flattening, and cognitive deficits that lead to persistent impairment of social adaptation and working capacity in young patients (Charlson et al., 2018; Marder & Cannon, 2019; Serafini et al., 2011). We suspect that acute polymorphic psychotic disorder and schizotypal disorder, which we are studying, may have identical pathological natures (Malhotra, Sahoo & Balachander, 2019; Lewine & Hart, 2020; Driver et al., 2020). They are characterized by an acute beginning of psychotic symptoms (duration less than two weeks) and are often connected with stress events and with rapid reduction of symptoms. Although if we study the pathogenetic of schizotypal disorder more deeply, we find that molecular hypotheses of schizophrenia’s origin, such as the endocannabinoid, kynurenine and dysontogenic theories, may be quite useful. The appearances of crossed pathogenetic sites of schizophrenia and schizotypal disorder onset have been studied (Ma et al., 2016). The pathogenesis of acute polymorphic psychotic disorder is connected with processes that are broken in schizophrenia patients. There are significant results that prove the stress diathesis hypothesis. This postulates that acute polymorphic psychotic disorder can manifest in persons with genetic vulnerabilities (Das et al., 2001). Moreover, López-Díaz, Lara & Luis Fernández-González (2018) suggested that patients with acute polymorphic psychotic disorder can have schizophrenia with their first episode.

Schizotypal disorder is characterized by eccentric behavior, thought disorder, and effects that resemble those seen in schizophrenia, but the severity of symptoms indicates no definite and characteristic schizophrenic anomalies. It is thought that individuals with schizotypal disorder share genetic and neurocognitive abnormalities with patients with schizophrenia (Siever & Davis, 2004). Some studies offer information about the appearance of non-specific symptoms in persons with schizotypal disorder. These symptoms may have a mixed nature of manifestation, with both genetic and environmental causes (Tsuang et al., 2002; Torgersen et al., 2002). Non-specificity in symptoms indicates that a single biological basis can underly different phenotypes and different biological bases can result in a single phenotype.

The main obstacle to developing highly effective treatment tactics is the lack of diagnostic criteria based on up-to-date data on the etiology and pathogenesis of mental disorders (Faiad et al., 2018). Currently, proteome studies provide the most information about the biological functioning of the body.

There are very few studies in the literature on the comparative proteomic analysis of different types of schizophrenic spectrum disorders. In addition, there are no articles specifically devoted to comparing proteomes in patients with simple schizophrenia and schizotypal disorder. It was only possible to discover a few early works on the study of APPD. Biochemical studies have revealed metabolic changes in amino acid pathways (Pepplinkhuizen et al., 2003) and increased levels of bilirubin in patients with APPD compared to patients with schizophrenia (Bach, Kindler & Strik, 2010; Pillmann et al., 2001).

In a recent study aimed at analyzing the genetic correlations between the blood plasma proteome and mental disorders, a set of 15 plasma proteins was identified that showed an association only with schizophrenia (Mahadevan et al., 2017). It is assumed that the identification of proteome features in mental patients may be complicated by multilevel interactions between changes caused by the pathological process and changes resulting from drug therapy. The question of whether biological interpretations based only on genetic information will reflect the fundamental genetic architecture of the most complex human traits has not yet been resolved. The problem of creating a panel of markers for mental disorders requires a systems biology approach (integration of genomic, transcriptomic and proteomic data, metabolomics, gene networks, epigenetics and environmental factors), where gene expression profiling is an important component of gene networks (Cheng et al., 2020).

In this article, methods of systems biology, classes of proteins, and molecular processes that specifically characterize disorders of the schizophrenia spectrum such as simple and paranoid schizophrenia, schizotypal disorder, and acute polymorphic psychotic disorder will be analyzed.

## Materials & Methods

The formation of patient groups for research and clinical verification of diagnoses was performed in the Department of Endogenous Disorders of the Mental Health Research Institute at the Tomsk National Research Medical Center of the Russian Academy of Sciences.

The study was conducted according to the guidelines of the Declaration of Helsinki and approved by the Ethical Committee of the Mental Health Research Institute No 151 from 14 March 2022 (No 151/1.2022).

The clinical verification of diagnoses was carried out by qualified psychiatrists according to the clinical criteria approved by the International Classification of Diseases, 10th Revision (ICD-10), for disorders of heading F2.

Criteria for inclusion of patients: male and female individuals aged 18 to 55 years; the diagnosis determined by psychiatrists; the presence of a signed form of informed consent to participate in the study.

Criteria for the inclusion of healthy individuals: conformity by gender and age with the studied group of patients; the presence of a signed form of informed consent to participate in the study.

Criteria for non-inclusion for all individuals: age under 18 and over 55 years; the presence of acute or exacerbated chronic infections, inflammatory or autoimmune diseases at the time of examination; acute infectious diseases less than four weeks before the start of the study; use of medications and narcotic substances; the presence of comorbid mental and neurological diseases in the patient; the presence of sexually transmitted diseases; refusal to participate in the study.

Comparative proteomic analysis was carried out on a sample of 35 people (18 women, 17 men). As part of the study, the following study groups were formed —simple and paranoid schizophrenia, schizotypal disorder, and acute polymorphic psychotic disorder (APPD).

Patients in the schizotypal disorder and APPD groups had not previously taken drug therapy, and patients with schizophrenia took a break from antipsychotic therapy for 2–3 months to a year.

The median age of all individuals included in the survey and the median duration of the illness for individuals with schizophrenia spectrum disorders are presented in Table 1. When the groups were compared pairwise, significant differences were identified between the ages of the control group and the schizotypal disorder (*p* = 0,004) and acute polymorphic psychotic disorder (*p* = 0,018) groups. When we compared the duration of illness in the simple schizophrenia group with the paranoid schizophrenia (*p* = 0,04) and APPD (*p* = 0,03) groups, significant differences were identified. Differences in sex and age between other groups were not significant.

**Table 1:** Characteristics of the individuals included in the study.

Group		Gender M/F	Age (years)	Duration of illness (years)
Schizophrenia	Simple schizophrenia	3/2	31.0 [25.5;40.5]	12.5 [6.5;17.00]
	Paranoid schizophrenia	6/6	30.5 [25.5;43.5]	3.0 [0.5;5.0]
Schizotypal disorder		3/2	19.0 [19.0;21.0]	2.5 [3.0;4.0]
Acute polymorphic psychotic disorder		2/3	24.0 [22.0;27.5]	0.03 [0.02;0.04]
Controls		3/8	39 [29;43]	—

### Sample preparation

Blood was collected in test tubes (Becton Dickinson Vacutainer, Netherlands) containing a clot activator. The blood was centrifuged to obtain serum. For 20 min at 2000 ×g using the Digicen 21R centrifuge (Orto Alresa, Madrid, Spain). Aliquots of serum was stored at −80 °C.

Serum samples were diluted 3-fold with sodium–phosphate buffer (phosphate buffered saline, PBS), centrifuged at 16,000 rpm for 1 min on a Centrifuge Z 36 HK centrifuge (Hermle labortechnik Gmbh, Wehingen, Germany) at 4 °C, and filtered through a standard Filtropur S filter (Sarstedt, Nümbrecht, Germany) with a diameter of 22 µm. Affinity depletion of high-abundant proteins (albumin, IgG, antitrypsin, IgA, transferrin, haptoglobin, fibrinogen, alpha2-macroglobulin, alpha1-acid glycoprotein, IgM, apolipoprotein AI, apolipoprotein AII, complement C3 and transthyretin) from samples were made on a chromatographic column Multiple Affinity Removal Column Human-14, 4.6 ×100 mm (Agilent, USA), using a chromatograph from the company ÄKTA pure (GE Healthcare, Chicago, IL, USA).q2 The purified protein mixture was concentrated by ultrafiltration through Amicon Ultra−0.5 mL kDa centrifuge filters (Merck Millipore, Molsheim, Alsace, France) in accordance with the protocol provided by the manufacturer. Protein concentration was measured by absorbance at 280/260 nm using an Epoch microplate spectrophotometer (BioTek, USA) with the software installed.

### Sample preparation: One-dimensional Laemmli PAG electrophoresis. Trypsinolysis

Electrophoretic separation (SDS-PAGE-based method) of serum proteins was carried out on a 1 mm thick 16 × 16 cm gel prepared with 12% acrylamide. A 20 µg portion of total protein in a Laemmli sample buffer was loaded into a 10 mm well of the gel and separated using a Protean II xi Cell (Bio-Rad, Hercules, CA, USA) device at 150–180 V. After protein separation, gels were stained with Coomassie Brilliant Blue G250, then washed the color in a solution of 70% ethanol for 2 h. Prior to trypsinolysis, the gel pieces were incubated three times in 50% (v/v) acetonitrile and 100 mM ammonium bicarbonate (pH 8.9) for 20 min. Then the gel pieces were dried in a vacuum concentrator. In-gel trypsinolysis of the proteins was carried out using Sequencing Grade Modified Trypsin (#V511A; Promega, Madison, WI, USA). Trypsin was added to each sample at a concentration of 0.01–0.025 µg/mL. Samples were incubated with trypsin at 37 °C for 18 h. The extraction of peptide mixtures from the gels was done with 50% acetonitrile in 5% formic acid, the procedure was triplicated. The extracts were lyophilized and frozen.

### Mass spectrometry analysis and protein identification

Mass spectrometric analysis was carried out in accordance with the previously described protocols of our studies and those of our colleagues (Naryzhny et al., 2016; Smirnova et al., 2019). The peptide samples obtained were analyzed using the Agilent HPLC system1100 Series (Agilent Technologies, Santa Clara, CA, USA) connected to LTQ Orbitrap Velos, equipped with a nanoelectrospray ion source (Thermo Scientific, Waltham, MA, USA). Peptide separations were carried out on an RP-HPLC Zorbax 300SB-C18 column (75 µm inner diameter and 150 mm length; Agilent Technologies, Santa Clara, CA, USA) using a linear gradient from 95% solvent A (water, 0.1% formic acid) and 5% solvent B (water, 0.1% formic acid, and 80% acetonitrile) to 60% solvent B over 85 min at a flow rate of 0.3 µL/min.

Mass spectra were acquired in the positive ion mode in a range of 300–1500 m/z with a resolution of 30,000 (m/z = 400) for MS and in the range from 100 m/z to m/z value determined by a charge state of the precursor at resolution 7,500 (m/z = 400) for MS/MS scans. The maximum integration time was 50 ms and 110 ms for precursor and fragment ions, correspondently. AGC target for precursor and fragment ions were set to 1*106 and 1*105, correspondently. An isolation intensity threshold of 5,000 counts was determined for precursor’s selection and up to top 10 precursors were chosen for fragmentation with high-energy collisional dissociation (HCD) at 35 eV. Precursors with a charge state of +1 and more than +5 were rejected and all measured precursors were dynamically excluded from triggering of a subsequent MS/MS for 60 s (Naryzhny et al., 2016; Smirnova et al., 2019).

Proteins were identified using the MASCOT software (http://www.matrixscience.com) in the UniProtKB database, *Homo sapiens* taxon. The search parameters have been set: the enzyme is trypsin, the tolerance for the monoisotopic peptide is ±10 ppm, the MS/MS tolerance window was set to ±0.01 Da, and one missed cleavage was allowed. Cysteine propionamide modification and oxidized methionine were chosen as variable modifications. The criteria of positive identification were set as following: peptide and protein FDR < 0.01, at least two matched peptides per protein, minimum protein score more than 30.

### Statistical and bioinformatics analysis

Statistical environment R was used to carry out statistical processing of the obtained results (R Core Team, 2018). Statistical differences were determined by Fisher’s exact test with Yates’s correction.

The Gene Ontology Database was used for the identification and analysis of biological pathways (http://geneontology.org/) (Gene Ontology Consortium, 2021). The PANTHER (Protein Analysis Through Evolutionary Relationships) tool was used to work with the Gene Ontology Database (http://pantherdb.org/) (Mi et al., 2019).

Proteins were classified into three categories by GO annotation: biological processes, regulation of biological processes, and molecular functions. For each category, a two-tailed Fisher’s exact test with FDR-controlling was used to measure the enrichment of differentially abundant proteins for all identified proteins.

## Results

**Figure 1:**
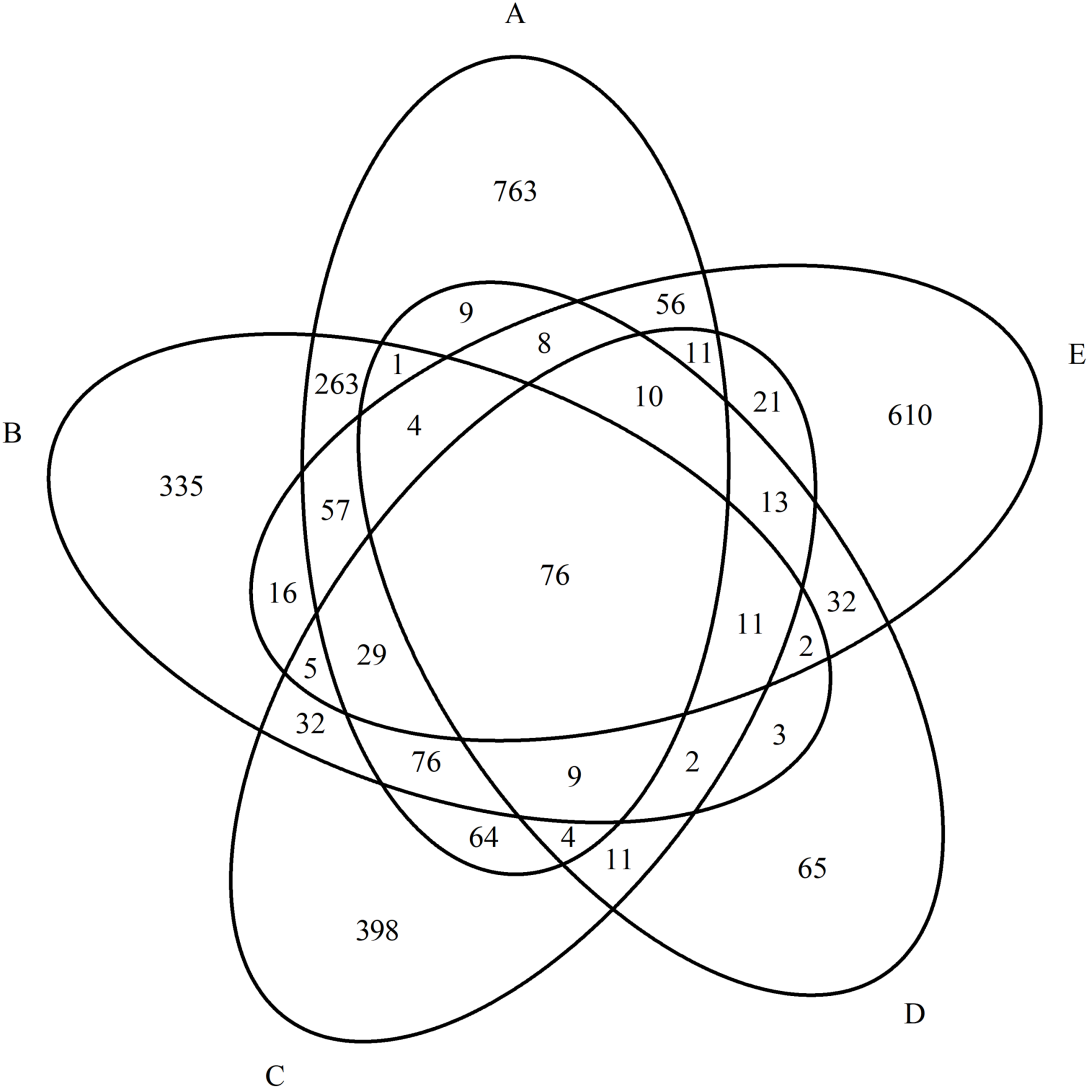
Diagram of the distribution of identified proteins between experimental groups. (A) Paranoid schizophrenia. (B) Schizotypal disorder. (C) Simple schizophrenia. (D) Acute polymorphic psychotic disorder. (E) Control group.

As a result of quantitative mass spectrometric analysis of a peptide mixture from purified blood serum in the MS and MS/MS mode and subsequent identification of proteins using the Mascot Version 2.1 software and the UniProtKB database, a total of 4,354 proteins were identified for all groups. We identified 961 proteins in the control group, 1,440 proteins in the paranoid schizophrenia group, 772 proteins in the simple schizophrenia group, 921 proteins in the schizotypal disorder group, and 260 proteins in the acute polymorphic psychotic disorder group. The overlaps between proteins in all groups are presented a Venn diagram in Fig. 1.

Subsequently, the results were subjected to statistical processing using Fisher’s exact test with Yates’s correction in the statistical environment R. This statistical approach made it possible to preserve the minor proteins in the blood serum in very low concentrations in the analysis results.

After statistical processing of the data, highly significant differences were revealed between the blood serum proteins of patients with schizophrenic spectrum disorders and the control group (Table 2). The smallest number of highly significant differences in the protein profiles were found in patients with acute polymorphic psychotic disorder compared with healthy people.

At a later stage, a statistical evaluation of the differences in serum proteomes among the studied groups of patients was carried out. Table 3 presents the results of an intergroup comparison of proteomic profiles. Significant differences were revealed among all groups.

**Figure 2:**
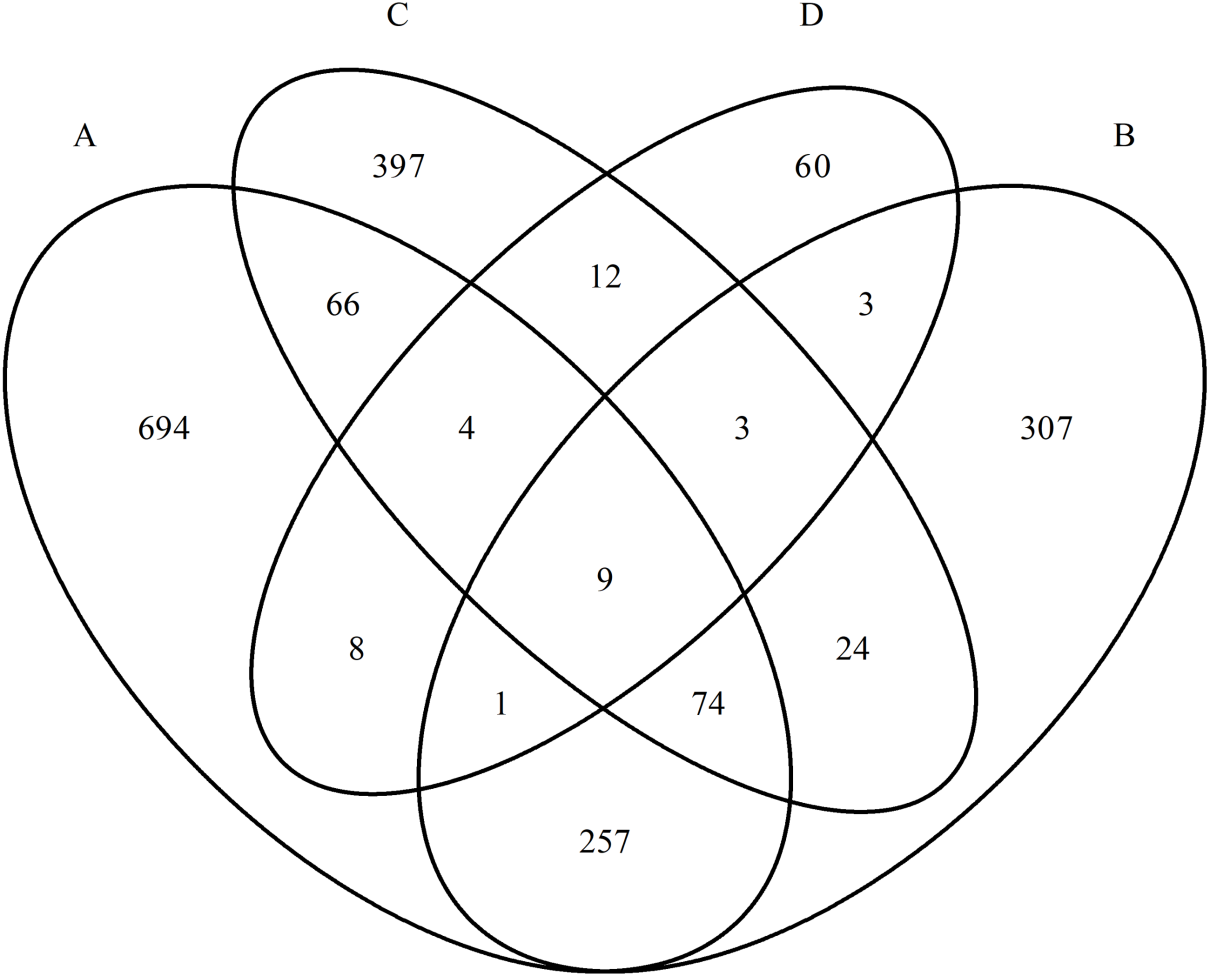
Diagram of the distribution of group-specific proteomic profiles between experimental groups. (A) Paranoid schizophrenia. (B) Schizotypal disorder. (C) Simple schizophrenia. (D) Acute polymorphic psychotic disorder.

Thus, despite one nosological group, the studied mental disorders showed a high specificity of proteomic profiles. Venn diagrams well reflect that there are few overlapping proteins in the studied groups (Fig. 2). Further, this is also confirmed by statistical analysis.

The most significant differences were found between the proteomic profiles of patients with paranoid schizophrenia and schizotypal disorder (*p* = 3.20E−12), and the least were found between patients with simple schizophrenia and schizotypal disorder (*p* = 0.016), which reflected the similarity in the first case and the differences in clinical and pathogenetic patterns in the second.

To further evaluate the differences in the proteomic profiles of the studied groups, proteins that occurred only in patients with one disorder and not in healthy individuals were selected. Unique protein spectra were formed by symmetric comparison of the proteomic profiles of all five groups with the removal of common proteins. The number of proteins in the paranoid schizophrenia group was 713, in the simple schizophrenia group 385 proteins, in the schizotypal disorder group 328 proteins, and in the acute polymorphic psychotic disorder group 60 proteins.

Then, an analysis of the functional activity of proteins was carried out on the unique protein spectra formed for each disorder. For each studied disorder, functional classes of proteins were determined using the PANTHER ™ Protein Class program (version 16.0, from 1 December 2020). Table 4 shows the number of proteins belonging to a certain class in each study group and indicates the level of significance of differences (according to the PANTHER program). Statistical analysis was carried out with Fisher’s exact test with false discovery rate-controlling.

**Table 2:** Differences between the proteomes of patients with schizophrenic spectrum disorders and healthy people, revealed by Fisher’s test with Yates’s correction (*p*, level).

Group	Fisher’s test, *p*
Control/Paranoid schizophrenia	9.67E−09
Control/Simple schizophrenia	9.65E−05
Control/Schizotypal disorder	0.0006
Control/Acute polymorphic psychotic disorder	6.35E−27

**Table 3:** Differences in proteomes among different groups of patients with schizophrenic spectrum disorders identified using Fisher’s test with Yates’s correction (*p*, level).

Group	Fisher’s test, *p*
Paranoid schizophrenia/Simple schizophrenia	0.0008
Paranoid schizophrenia/Schizotypal disorder	3.20E−12
Paranoid schizophrenia/Acute polymorphic psychotic disorder	0.006
Simple schizophrenia/Schizotypal disorder	0.016
Simple schizophrenia/Acute polymorphic psychotic disorder	5.35E−09
Schizotypal disorder/Acute polymorphic psychotic disorder	2.06E−06

**Table 4:** Protein classes were determined using the PANTHER program for each study group (Fisher’s exact test with FDR).

**Protein class**	**Paranoid** **schizophrenia**		**Simple** **schizophrenia**		**Schizotypal** **disorder**		**Acute polymorphic psychotic disorder**	
	No^*^	*P*-value	No^*^	*P*-value	No^*^	*P*-value	No^*^	*P*-value
Extracellular matrix protein			12	5.84E−03				
Protein-binding activity modulator	63	3.8E−03	11	3.41E−02				
Vesicle coat protein			4	4.69E−02				
Nucleic acid metabolism protein	32	4.79E−02					12	4.68E−02
Metabolite interconversion enzyme	101	2.64E−02	20	1.11E−02	75	3.88E−02		
Protein modifying enzyme			5	3.12E−02	27	4.50E−02	5	2.14E−02
Gene-specific transcriptional regulator	60	2.06E−02	12	4.22E−02				
Translational protein	83	2.55E−05						
Transmembrane signal receptor	15	3.00E−03	18	2.24E−02	4	2.04E−02	4	1.03E−02
Transporter							5	2.33E−02
Chromatin/chromatin-binding, or -regulatory protein	25	1.07E−02					2	1.9E−02
Cytoskeletal protein	83	3.11E−06	59	6.05E−03	23	4.32E−02		
Chaperone					3	2.20E−02		

**Notes.**

* No, Number of proteins.

During our research, when studying the distribution of proteins by class in different disorders of the schizophrenia spectrum, interesting and unique results were obtained. Thus, most of the proteins mediating translational and transcriptional processes were found in patients with paranoid schizophrenia. For the group with simple schizophrenia, the main number of proteins were related to the receptor apparatus, vesicular transport, and extracellular matrix proteins. The proteins of patients with schizotypal disorder mostly coincided with the classes of other groups of patients, except of chaperone proteins, which were not found in any other disorder. The main class of proteins identified in patients with an acute polymorphic psychotic disorder metabolized nucleic acids.

To further study the properties of protein profiles in each study group, the biological processes carried out by the identified proteins were also determined using PANTHER ™ GO slim. The tables below show the biological processes characteristic of each studied group of disorders. In addition data about processes for men and women are presented, in which it is clear that the differences do not depend on gender (Table S1). In all groups, the processes in men and women coincide in 50–60%.

In the group with paranoid schizophrenia, 713 proteins were involved in 105 bio-logical processes combined into groups (Table 5). Biological processes characterizing significant proteins in the blood serum of patients with paranoid schizophrenia (PANTHER).

**Table 5:** Biological processes characterizing significant proteins in the blood serum of patients with paranoid schizophrenia (PANTHER).

	Groups of biological processes	Number of processes	Process ID^*^
Metabolic processes	Cellular macromolecule biosynthetic process	2	GO:0034645
	Macromolecule metabolic process	4	GO:0043170
	Nucleic acid metabolic process	8	GO:0090304
	Metabolism of nitrogenous compounds	11	GO:0006807
Cellular processes	Cell population proliferation	1	GO:0008283
	Vacuole organization	1	GO:0007033
	Intracellular transport	3	GO:0046907
	Chromosome organization	4	GO:0051276
	Actin cytoskeleton organization	5	GO:0030036
	Cellular protein localization	5	GO:0034613
	Ribosome biogenesis	6	GO:0042254
	Cellular component assembly	7	GO:0022607
	Cellular component organization	10	GO:0016043
Regulation of biological processes	Regulation of cellular amide metabolic process	1	GO:0034248
	Regulation of cellular component size	2	GO:0032535
	Intracellular signal transduction	2	GO:0035556
	Regulation of RNA metabolic process	2	GO:0051252
	Regulation of chromatin organization	2	GO:1902275
	Regulation of programmed cell death	2	GO:0043067
	Regulation of protein metabolic process	4	GO:0051246
	Positive regulation of actin filament polymerization	4	GO:0030838
	Regulation of actin cytoskeleton organization	6	GO:0032956
	Regulation of cellular component organization	6	GO:0051128
	Regulation of gene expression	7	GO:0010468

**Notes.**

* Process ID in Gene Ontology Database.

In patients with paranoid schizophrenia, most of the identified proteins were associated with the biosynthesis and assembly of macromolecular complexes, as well as subsequent cell transport and the location of organelles inside the cell. A large group of proteins was associated with the synthesis, functioning, and regulation of the actin cytoskeleton. Proteins involved in various regulatory processes, in particular, the regulation of translation and transcription processes, were identified.

In the group with simple schizophrenia, 385 proteins were involved in 50 biological processes (Table 6).

**Table 6:** Biological processes characterizing significant proteins in the blood serum of patients with simple schizophrenia (PANTHER).

	Groups of biological processes	Number of processes	Process ID^*^
Metabolic processes	Macromolecule catabolic process	1	GO:0009057
Cellular processes	Movement of cell or subcellular component	1	GO:0006928
	Cytoskeleton-dependent intracellular transport	2	GO:0030705
	Cell cycle process	2	GO:0022402
	Protein localization to endoplasmic reticulum	3	GO:0070972
	Vesicle-mediated transport	5	GO:0016192
	Cellular component organization	8	GO:0016043
Regulation of biological processes	Cell–substrate adhesion	1	GO:0031589
	Regulation of wound healing	1	GO:0061041
	Negative regulation of catalytic activity	1	GO:0043086
	Positive regulation of metabolic process	1	GO:0009893
	Regulation of signal transduction	3	GO:0009966
	Regulation of lipase activity	3	GO:0060191
	Regulation of transport	4	GO:0051049
	Regulation of phospholipase activity	5	GO:0010517
	Regulation of immune response	9	GO:0050776

**Notes.**

* Process ID in Gene Ontology Database.

A significant number of proteins in patients with simple schizophrenia regulated the cell’s main metabolic and transport processes, and some proteins provided intercellular communication. In contrast to the widely represented metabolic processes in the paranoid schizophrenia group, the proteins of patients with simple schizophrenia mainly carried out catabolism of macromolecules. Common to simple and paranoid schizophrenia were the processes of organelle distribution within the cell and intracellular transport of molecules. However, for the group with simple schizophrenia, most of the transport proteins mediated the transfer of molecules and signals between cells. The processes included in the group of vesicular transport were associated with exocytosis and secretion.

In the group represented by patients with schizotypal disorder, 328 proteins were involved in 59 biological processes (Table 7).

**Table 7:** Biological processes characterizing significant proteins in the blood serum of patients with schizotypal disorder (PANTHER).

	Groups of biological processes	Number of processes	Process ID^*^
Metabolic processes	Cellular macromolecule metabolic process	1	GO:0044260
	Nucleic acid metabolic process	2	GO:0090304
	fatty acid metabolic process	2	GO:0006631
	Ribonucleotide metabolic process	4	GO:0009259
	Organonitrogen compound metabolic process	4	GO:1901564
	Metabolic process	7	GO:0008152
	Biosynthetic process	8	GO:0009058
Cellular processes	Cellular iron ion homeostasis	1	GO:0006879
	Synaptic vesicle cycle	2	GO:0099504
	Chaperone-mediated protein folding	2	GO:0061077
	Production of molecular mediator of immune response	2	GO:0002440
	Developmental process	2	GO:0032502
	Vesicle-mediated transport	3	GO:0016192
	Cellular component organization	3	GO:0016043
Regulation of biological processes	Regulation of response to external stimulus	1	GO:0032101
	Regulation of neuron differentiation	1	GO:0045664
	G protein-coupled receptor signaling pathway	1	GO:0007186
	Ionotropic glutamate receptor signaling pathway	1	GO:0035235
	Regulation of protein stability	2	GO:0031647
	Regulation of response to stimulus	2	GO:0048583
	Regulation of neurotransmitter receptor activity	2	GO:0099601
	Regulation of metabolic process	6	GO:0019222

**Notes.**

* Process ID in Gene Ontology Database.

Proteins in the schizotypal disorder group were associated with a large number of metabolic processes and provided high synthetic activity of the cell. Metabolic processes coincided for the schizotypal disorder and paranoid schizophrenia groups but were represented by a wider variety, for example, the presence of purine base synthesis. Also, many proteins were associated with the catabolic processes of amino acids, organic acids, and nucleotides. Catabolic processes were also characteristic of proteins from the simple schizophrenia group. Proteins that characterized cellular processes differed significantly from the profiles of other patients and were mainly represented by the transport of vesicles in the synapse, the formation of synaptic endings, and the development of individual brain structures. In the blood serum of patients with schizotypal disorder, specific chaperone proteins were also detected that were absent in other groups. The regulatory proteins found in these patients ensured the functioning of cellular process proteins and regulated the differentiation of neurons, the activity of glutamate receptors, synaptic signal transmission, and metabolic processes in the cell.

In the group with acute polymorphic psychotic disorder, 60 proteins were involved in 33 biological processes (Table 8).

**Table 8:** Biological processes characterizing significant proteins in the blood serum of patients with acute polymorphic psychotic disorder (PANTHER).

	Groups of biological processes	Number of processes	Process ID^*^
Cellular processes	Antigen processing and presentation	1	GO:0019882
	Chromosome organization	1	GO:0051276
	Sequestering of calcium ion	1	GO:0051208
	Myelination	1	GO:0042552
	Transmembrane transport	2	GO:0055085
	Cell death	5	GO:0008219
Regulation of biological processes	Regulation of cytosolic calcium ion concentration	1	GO:0051480
	Negative regulation of T cell proliferation	1	GO:0042130
	Positive regulation of proteolysis	1	GO:0045862
	Regulation of B cell proliferation	1	GO:0030888
	Regulation of cell cycle process	1	GO:0010564
	Negative regulation of signal transduction	1	GO:0009968
	Regulation of system process	3	GO:0044057
	Regulation of sequestering of calcium ion	3	GO:0051282
	Regulation of programmed cell death	4	GO:0043067
	Regulation of hydrolase activity	6	GO:0051336

**Notes.**

* Process ID in Gene Ontology Database.

The proteins responsible for the biological processes in the acute polymorphic psychotic disorder group differed significantly from the proteins that characterized the processes of all other groups. These were mainly proteins associated with the processes of cell death, including apoptosis of neurons. However, there were also proteins associated with the process of myelination and the organization of chromosomes. Cell death is regulated by a large number of proteins, including proteins that increase the activity of hydrolases and proteases. The processes that inhibit neuronal signal transmission were significantly expressed. Proteins mediating cellular metabolism for these patients did not show significant differences from the other groups of patients.

## Discussion

In the presented work, a comparative proteomic analysis of the blood sera of schizophrenia spectrum disorders was performed. When comparing the proteomic profiles of patients with paranoid and simple schizophrenia, acute polymorphic psychotic disorder (APPD), and schizotypal disorder with the proteomic profile of healthy individuals, highly significant differences were revealed among all groups. The smallest number of highly significant differences in protein profiles compared with the control group were found in patients with APPD (*p* = 0.00635). Usually, such a diagnosis is made for people with a first psychotic episode, and the outcome can be twofold: either a complete recovery or the development of schizophrenia. The smallest number of differences in the proteomes of these patients was from the short duration of the disease and indicated minimal pathological changes in their organisms (Gehlenborg et al., 2010).

When comparing the proteomic profiles of the blood sera of all the studied groups of patients, highly significant statistical differences were also obtained. The greatest number of differences in the protein profiles were revealed between patients with paranoid schizophrenia and schizotypal disorder. From a clinical point of view, these patients also differed the most from each other. The smallest number of differences in the proteomic profiles of patients were found between patients with paranoid schizophrenia and acute polymorphic psychotic disorder, and (minimal differences) between patients with simple schizophrenia and schizotypal disorder. Patients with an acute polymorphic psychotic disorder are very often subsequently diagnosed with paranoid schizophrenia, and the resulting picture of the comparison of proteomes indicated the common pathogenetic changes in these disorders. Simple schizophrenia is often similar in its clinical manifestations to a schizotypal disorder, and changes in the protein spectrum also confirmed the similarity on the biochemical level (Vora et al., 2018).

With the help of the international program PANTHER ™ GO slim, the main functional classes of proteins for each studied disorder were determined. Even though most proteins in all groups were distributed between three main classes: metabolic enzymes, cytoskeleton, and transmembrane signaling receptors as a result of this comparison, unique results were obtained for each group of disorders. The data obtained to a certain extent coincided with those presented in the literature (Woods et al., 2012; Perkovic et al., 2017; Mahadevan et al., 2017). However, in the studied literature, the detected proteins were not classified according to their functions and their relationship with pathological mechanisms. According to the proteins that showed significant differences among all the studied groups, patients with paranoid schizophrenia were characterized by the main classes of proteins responsible for translation and transcription processes (about 200 proteins); for the group of simple schizophrenia—proteins of the receptor apparatus, extracellular matrix, and vesicular transport (about 100 proteins); for patients with the schizotypal disorder—chaperone proteins, which are unique to this type of disorder, and metabolic enzymes (75 proteins). The proteins of patients with an acute polymorphic psychotic disorder were mostly classified as proteins that metabolize nucleic acids. Proteins of this category were found only in this group of patients and in the paranoid schizophrenia group. Chromatin-binding/regulating proteins were also found only in these two groups. This result suggested that for these groups of patients, the prevalence of proteins of the synthetic cell apparatus reflects active intracellular processes, which can be justified by the activation of repair processes. It may indicate a favourable prognosis for the course of the disease (English et al., 2018).

This analysis suggested that in cases of patients with paranoid schizophrenia, repair processes prevail, which determines the possibility of remission in this group of patients. The protein classes that predominate in the blood serum of patients with simple schizophrenia probably indicate a more severe course of the pathological process, reflected on the cellular level. Schizotypal disorder, characterized by the presence of such classes of proteins as chaperones, which appear when the body reacts to extreme exposure, speaks primarily to the highly adaptive abilities of the system with this disorder (Xie et al., 2020). The body’s response to psychogenic stress, in acute psychosis, is also characterized by a class of proteins identified in patients with an acute polymorphic psychotic disorder.

For further analysis of the proteins of each group of disorders, specific biological processes were identified using the program PANTHER again.

As a result of the undertaken research, the processes that characterized each disease were determined. In patients with paranoid schizophrenia, most of the identified proteins were involved in the processes of biosynthesis, cell transport, and the location of organelles inside the cell, that is, in the cell structure formation. A large group of proteins was involved in the processes of synthesis, functioning, and regulation of the actin cytoskeleton. The identified proteins were involved in 15 mechanisms of translation and transcription regulation. A significant number of proteins that characterized the group of patients with simple schizophrenia regulated the cell’s main metabolic and transport processes. In contrast to the group with paranoid schizophrenia, in which the processes of anabolism predominated, the proteins of patients with simple schizophrenia were mainly involved in the processes of catabolism. The processes of intracellular transport and distribution of organelles, as well as the growth and development of neurons, were actively occurring processes in both simple and paranoid schizophrenia. However, in the group with simple schizophrenia, most of the transport proteins mediated the transmission of signaling molecules between cells, receptor transmission, and its regulation. Most proteins in the group of patients with the schizotypal disorder represented anabolic processes and, thereby, provided the high synthetic activity of the cell. Some of the metabolic processes coincided with similar ones in patients with paranoid schizophrenia but were widely represented, such as purine base synthesis. Proteins that characterized cellular processes in patients with schizotypal disorder were mainly represented by the transport of vesicles in the synapse, the formation of synaptic endings, and the processes of neurogenesis. In the blood sera of patients with schizotypal disorder, specific chaperone proteins were also detected that were not found in other studied groups. The biological processes presented in the group with acute polymorphic psychotic disorder differed significantly from the proteins that characterized the processes of all other groups. Many active processes involved in cell death, including apoptosis of neurons, were found in patients with APPD, where proteins associated with the process of myelination and the organization of chromosomes were found. All these processes cause acute inflammation, which characterizes acute psychosis in cases of APPD. Thus, the processes specific to each type of studied disorder and characterizing the pathogenetic and biochemical features of the course of the disease were identified.

## Conclusions

Biological psychiatry has mainly been focused on the study of the relationship between quantitative changes in individual indicators of a particular disorder. Using the proteomics method, it was possible to identify the classes of proteins and molecular processes that most likely characterize some of the most common disorders of the schizophrenic spectrum. This research provides evidence from the point of view of systems biology for existing significant differences in biological pathways and molecular mechanisms among schizophrenia spectrum disorders. According to the proteins that showed significant differences among all the studied groups, we concluded that the main classes of proteins responsible for translation and transcription processes are characteristic for patients with paranoid schizophrenia. These results proved that the processes of repair prevail in these patients, which leads to the possibility of remission for this group. A significant part of the proteome of patients with simple schizophrenia referred to proteins that regulate the cell’s main metabolic and transport processes. However, in contrast to the widely presented metabolic processes in patients with paranoid schizophrenia, the proteins of patients with simple schizophrenia mainly regulated the processes of catabolism. The processes of organelle distribution and intracellular transport were common to both forms of schizophrenia. For the simple schizophrenia group, most of the transport proteins mediated receptor transmission and its regulation. These results mainly characterized the greater depth of the pathological process in this form of schizophrenia. The proteins of patients with schizotypal disorder mostly coincided with the classes of other groups of patients, except of chaperone proteins, which were not found in any other disorder. The proteins of patients with schizotypal disorder were associated with a large number of metabolic processes and provided high synthetic activity of the cell. The metabolic processes of patients with schizotypal disorder and paranoid schizophrenia partially coincided but were represented by a wide variety. In addition, with this disorder, many proteins were associated with catabolic processes of amino acids, organic acids, and nucleotides and coincided with those in patients with the simple form of schizophrenia, which correlated with some common clinical manifestations of these disorders. These results suggested that, with further study, chaperone proteins can be a specific marker of the schizotypal disorder. The proteins of patients with an acute polymorphic psychotic disorder were mostly classified as proteins that metabolized nucleic acids. Proteins of this category were found only in these patients and in paranoid schizophrenia. This suggested that for these patients, the prevalence of proteins of the synthetic cell apparatus could be justified by the activation of repair processes and may indicate a favourable prognosis of the course of the disease. Moreover, a large number of active processes involved in cell death, including apoptosis of neurons, were found in patients with APPD, including proteins associated with the process of myelination and the organization of chromosomes. All these processes cause acute inflammation, which characterizes acute psychosis in cases of APPD. These new results will form the basic knowledge about the molecular mechanisms of the pathogenesis of these disorders.

However, it will be possible to speak with complete confidence about the discovered mechanisms and the proteins involved in them after confirming these results in a larger number of patients and a detailed study of the effect of drug therapy on these processes. The results obtained may be of interest when studying the pathophysiology and molecular mechanisms of disorders of the schizophrenic spectrum.

## Limitations

This study has several limitations. First, the group of patients with the studied dis-eases was small. Serum proteomic studies are very expensive and time-consuming studies. High-quality screening analysis of the proteomic composition of blood serum analyses a huge number of peptides, identifying thousands of proteins by short ones. The total number of identified proteins in this study was 4,354. With an increase in the number of patients in the groups, not only does the cost of work increase significantly, but the volume of processed data also increases significantly with each sample. Therefore, in this type of proteomic analysis, we tried to use the minimum possible number of people in the groups. Second, this study took place in a relatively short period, with consecutive patient recruitment. The cross-sectional design of this pilot study limited the mechanistic interpretation of inferred temporal changes, particularly in relation to such factors as age, gender, and disease duration. Therefore, the sex groups were randomly formed and emphasized the fact that men are predominantly affected by mental disorders. We conducted additional studies and investigated whether the obtained data were gender dependent and presented the results in additional materials. We did not find such a connection. A significant difference in the ages of patients with APPD and schizotypal disorder with control resulted from the early age of manifestation of these disorders. Third, the group of patients with simple schizophrenia had a significantly longer duration of the disease in comparison with other groups. However, the fact that negative symptoms and simple schizophrenia predominate in long term illness is well known (Fenton & McGlashan, 1991). There are various reasons for this. One of the reasons why psychiatrists cannot determine disease and correctly classify it by ICD-10 is the severity of symptoms that do not cross the “threshold” border. Finally, our ability to work and publish results was limited by the size and requirements of the RSF grant. In this article we published only a part of the results of our work. Using the methods of statistics and bioinformatics, the manuscript illustrates the processes and pathways that characterize each disease, which are very important for understanding the peculiarities of the pathogenesis of mental disorders. We will present full information about altered proteins in a separate article.

##  Supplemental Information

10.7717/peerj.13907/supp-1 Supplemental Information 1 Biological processes characterizing significant proteins in the blood serum of patients in all experimental groups with analysis stratified by sex (PANTHER)

## Additional Information and Declarations

### Data Availability

The following information was supplied regarding data availability: Raw data is available at Figshare: Dmitrieva, Elena (2022): Proteomic profile of serum from patients with schizophrenia spectrum disorders. figshare. Dataset. https://doi.org/10.6084/m9.figshare.19767709.v1.
