# Supplementary material for: Proteomic profile of serum from patients with schizophrenia spectrum disorders
Source: PeerJ. 2022 Aug 30;10:e13907. doi: 10.7717/peerj.13907 (PMC9438766; doi:10.7717/peerj.13907)
Supplement: Supplemental Information 1 [file peerj-10-13907-s001.docx]

Table S1:

Biological processes characterizing significant proteins in the blood serum of patients in all experimental groups with analysis stratified by sex (PANTHER).

Control group Male

|  | Groups of biological processes | Number of processes | Process ID * |
| --- | --- | --- | --- |
| Biological regulation | negative regulation of blood coagulation | 1 | GO:0030195 |
|  | regulation of catalytic activity | 15 | GO:0050790 |
|  | regulation of cell adhesion | 7 | GO:0030155 |
|  | regulation of signaling | 1 | GO:0023051 |
|  | regulation of transport | 5 | GO:0051049 |
|  | signal transduction | 6 | GO:0007165 |
| Metabolic process | macromolecule metabolic process | 7 | GO:0043170 |
|  | organonitrogen compound metabolic process | 5 | GO:1901564 |
| Cellular process | cell death | 2 | GO:0008219 |
|  | cellular component organization | 11 | GO:0016043 |
|  | multicellular organismal process | 4 | GO:0032501 |
|  | transport | 2 | GO:0006810 |

Control group Female

|  | Groups of biological processes | Number of processes | Process ID * |
| --- | --- | --- | --- |
| Biological regulation | regulation of catalytic activity | 10 | GO:0050790 |
|  | regulation of cell adhesion | 3 | GO:0030155 |
|  | regulation of cell cycle | 8 | GO:0051726 |
|  | regulation of metabolic process | 4 | GO:0019222 |
|  | regulation of organelle organization | 4 | GO:0033043 |
|  | regulation of transport | 2 | GO:0051049 |
| Metabolic process | cellular macromolecule biosynthetic process | 4 | GO:0034645 |
|  | macromolecule metabolic process | 3 | GO:0043170 |
| Cellular process | cell cycle process | 4 | GO:0022402 |
|  | cellular component organization | 8 | GO:0016043 |
|  | multicellular organismal process | 7 | GO:0032501 |

Acute polymorphic psychotic disorder Group

Male

|  | Groups of biological processes | Number of processes | Process ID * |
| --- | --- | --- | --- |
| Biological regulation | ion homeostasis | 1 | GO:0050801 |
|  | negative regulation of blood coagulation | 1 | GO:0030195 |
|  | positive regulation of gene expression | 1 | GO:0010628 |
|  | positive regulation of peptide secretion | 1 | GO:0002793 |
|  | positive regulation of response to stimulus | 1 | GO:0048584 |
|  | regulation of cell activation | 2 | GO:0050865 |
|  | regulation of cell adhesion | 2 | GO:0030155 |
|  | Regulation of cytosolic calcium ion concentration | 1 | GO:0051480 |
|  | Regulation of hydrolase activity | 1 | GO:0051336 |
|  | regulation of metabolic process | 2 | GO:0019222 |
|  | regulation of protein transport | 1 | GO:0051223 |
|  | regulation of signaling | 1 | GO:0023051 |
| Metabolic process | macromolecule metabolic process | 7 | GO:0043170 |
|  | organonitrogen compound metabolic process | 5 | GO:1901564 |
| Cellular process | cell death | 2 | GO:0008219 |
|  | cellular component organization | 11 | GO:0016043 |
|  | multicellular organismal process | 4 | GO:0032501 |
|  | transport | 2 | GO:0006810 |

Acute polymorphic psychotic disorder Group

Female

|  | Groups of biological processes | Number of processes | Process ID * |
| --- | --- | --- | --- |
| Biological regulation | positive regulation of metabolic process | 1 | GO:0009893 |
|  | regulation of gene expression | 2 | GO:0010468 |
|  | regulation of cell cycle process | 1 | GO:0010564 |
|  | positive regulation of gene expression | 1 | GO:0010628 |
|  | regulation of metabolic process | 3 | GO:0019222 |
|  | regulation of cell adhesion | 1 | GO:0030155 |
|  | regulation of chromosome organization | 2 | GO:0033044 |
|  | regulation of system process | 1 | GO:0044057 |
|  | positive regulation of cell cycle | 1 | GO:0045787 |
|  | positive regulation of biological process | 2 | GO:0048518 |
|  | regulation of transport | 2 | GO:0051049 |
|  | regulation of cellular component organization | 2 | GO:0051128 |
| Metabolic process | DNA metabolic process | 1 | GO:0006259 |
|  | DNA replication | 2 | GO:0006260 |
|  | nucleobase-containing compound metabolic  process | 2 | GO:0006139 |
|  | nucleoside phosphate metabolic process | 2 | GO:0006753 |
|  | organic substance metabolic process | 2 | GO:0071704 |
|  | organonitrogen compound metabolic process | 2 | GO:1901564 |
|  | purine-containing compound metabolic process | 2 | GO:0072521 |
|  | translational initiation | 1 | GO:0006413 |
| Cellular process | antigen processing and presentation | 1 | GO:0019882 |
|  | cell cycle process | 4 | GO:0022402 |
|  | cell death | 4 | GO:0008219 |
|  | cellular component organization | 6 | GO:0016043 |
|  | Chromosome organization | 1 | GO:0051276 |
|  | leukocyte activation | 4 | GO:0045321 |
|  | multicellular organismal process | 2 | GO:0032501 |
|  | organelle localization | 4 | GO:0051640 |
|  | protein folding | 2 | GO:0006457 |
|  | Transmembrane transport | 4 | GO:0055085 |
|  | transport | 2 | GO:0006810 |
|  | vesicle-mediated transport | 4 | GO:0016192 |

Schizotypal disorder Group

Male

|  | Groups of biological processes | Number of processes | Process ID * |
| --- | --- | --- | --- |
| Biological regulation | blood coagulation | 1 | GO:0007596 |
|  | negative regulation of blood coagulation | 1 | GO:0030195 |
|  | negative regulation of cellular catabolic process | 1 | GO:0031330 |
|  | organonitrogen compound metabolic process | 1 | GO:1901564 |
|  | positive regulation of cellular process | 1 | GO:0048522 |
|  | positive regulation of peptide secretion |  | GO:0002793 |
|  | regulation of catalytic activity | 1 | GO:0050790 |
|  | regulation of immune response | 1 | GO:0050776 |
|  | Regulation of response to external stimulus | 1 | GO:0032101 |
|  | regulation of signal transduction | 1 | GO:0009966 |
|  | G protein-coupled receptor signaling pathway | 1 | GO:0007186 |
| Metabolic process | organonitrogen compound metabolic process | 2 | GO:1901564 |
|  | translation | 1 | GO:0006412 |
|  | metabolic process | 3 | GO:0008152 |
| Cellular process | cell death | 1 | GO:0008219 |
|  | cellular component organization | 5 | GO:0016043 |
|  | cellular metabolic process | 1 | GO:0044237 |
|  | chaperone-mediated protein folding | 1 | GO:0061077 |
|  | Developmental process | 1 | GO:0032502 |
|  | gene expression | 1 | GO:0010467 |
|  | peptide secretion | 1 | GO:0002790 |
|  | protein folding | 1 | GO:0006457 |

Schizotypal disorder Group

Female

|  | Groups of biological processes | Number of processes | Process ID * |
| --- | --- | --- | --- |
| Biological regulation | blood coagulation | 1 | GO:0007596 |
|  | G protein-coupled receptor signaling pathway | 1 | GO:0007186 |
|  | regulation of catalytic activity | 1 | GO:0050790 |
|  | regulation of cell activation | 1 | GO:0050865 |
|  | regulation of cell cycle process | 1 | GO:0010564 |
|  | regulation of gene expression | 2 | GO:0010468 |
|  | regulation of metabolic process | 1 | GO:0019222 |
|  | regulation of protein stability | 1 | GO:0031647 |
|  | regulation of response to stimulus | 1 | GO:0048583 |
|  | response to external stimulus | 1 | GO:0009605 |
|  | response to stress | 2 | GO:0006950 |
|  | signal transduction | 1 | GO:0007165 |
|  | regulation of signal transduction | 2 | GO:0009966 |
| Metabolic process | immune response | 2 | GO:0006955 |
|  | immune system process | 2 | GO:0002376 |
|  | metabolic process | 2 | GO:0008152 |
|  | organonitrogen compound metabolic process | 1 | GO:1901564 |
| Cellular process | cell death | 1 | GO:0008219 |
|  | cell recognition | 1 | GO:0008037 |
|  | cellular component organization | 1 | GO:0016043 |
|  | cellular response to stress | 2 | GO:0033554 |
|  | chaperone-mediated protein folding | 1 | GO:0061077 |
|  | chromosome organization | 3 | GO:0051276 |
|  | Developmental process | 2 | GO:0032502 |
|  | ion transmembrane transport | 1 | GO:0034220 |
|  | multicellular organism development | 1 | GO:0007275 |
|  | multicellular organismal process | 1 | GO:0032501 |
|  | production of molecular mediator of immune  response | 2 | GO:0002440 |
|  | protein folding | 1 | GO:0006457 |
|  | vesicle-mediated transport | 3 | GO:0016192 |

Schizophrenia Group

Male

|  | Groups of biological processes | Number of processes | Process ID * |
| --- | --- | --- | --- |
| Biological regulation | hemostasis | 2 | GO:0007599 |
|  | intracellular signal transduction | 1 | GO:0035556 |
|  | multicellular organismal homeostasis | 2 | GO:0048871 |
|  | negative regulation of catalytic activity | 1 | GO:0043086 |
|  | positive regulation of T cell activation | 2 | GO:0050870 |
|  | regulation of actin cytoskeleton organization | 3 | GO:0032956 |
|  | regulation of catalytic activity | 5 | GO:0050790 |
|  | regulation of metabolic process | 3 | GO:0019222 |
|  | regulation of developmental process | 4 | GO:0050793 |
|  | regulation of gene expression | 2 | GO:0010468 |
|  | regulation of programmed cell death | 8 | GO:0043067 |
|  | regulation of protein stability | 4 | GO:0031647 |
|  | regulation of RNA metabolic process | 6 | GO:0051252 |
|  | regulation of secretion | 2 | GO:0051046 |
| Metabolic process | ATP metabolic process | 2 | GO:0046034 |
|  | cellular macromolecule catabolic process | 7 | GO:0044265 |
|  | cholesterol transport | 1 | GO:0030301 |
|  | multicellular organismal process | 6 | GO:0032501 |
|  | organonitrogen compound metabolic process | 3 | GO:1901564 |
|  | macromolecule metabolic process | 5 | GO:0043170 |
|  | nucleic acid metabolic process | 5 | GO:0090304 |
| Cellular process | actin cytoskeleton organization | 2 | GO:0030036 |
|  | axonemal dynein complex assembly | 1 | GO:0070286 |
|  | cell adhesion | 2 | GO:0007155 |
|  | cellular component organization | 3 | GO:0016043 |
|  | cellular macromolecule localization | 7 | GO:0070727 |
|  | chromosome organization | 5 | GO:0051276 |
|  | developmental process | 2 | GO:0032502 |
|  | immune response | 2 | GO:0006955 |
|  | movement of cell or subcellular component | 2 | GO:0006928 |
|  | protein folding | 12 | GO:0006457 |
|  | protein transport | 8 | GO:0015031 |
|  | response to external stimulus | 2 | GO:0009605 |
|  | transposition | 1 | GO:0032196 |
|  | vacuole organization | 4 | GO:0007033 |
|  | vesicle-mediated transport | 4 | GO:0016192 |

Schizophrenia Group

Female

|  | Groups of biological processes | Number of processes | Process ID * |
| --- | --- | --- | --- |
| Biological regulation | activation of immune response | 2 | GO:0002253 |
|  | cation homeostasis | 1 | GO:0055080 |
|  | cellular homeostasis | 2 | GO:0019725 |
|  | intracellular signal transduction | 3 | GO:0035556 |
|  | regulation of actin cytoskeleton organization | 7 | GO:0032956 |
|  | regulation of catalytic activity | 2 | GO:0050790 |
|  | regulation of cell activation | 7 | GO:0050865 |
|  | regulation of cellular response to stress | 5 | GO:0080135 |
|  | regulation of gene expression |  | GO:0010468 |
|  | regulation of metabolic process | 4 | GO:0019222 |
|  | regulation of multicellular organismal process | 8 | GO:0051239 |
|  | regulation of protein secretion | 6 | GO:0050708 |
|  | regulation of RNA metabolic process | 7 | GO:0051252 |
|  | regulation of sequestering of calcium ion | 2 | GO:0051282 |
|  | signal transduction | 1 | GO:0007165 |
| Metabolic process | macromolecule metabolic process | 15 | GO:0043170 |
|  | metabolism of nitrogenous compounds | 8 | GO:0006807 |
|  | multicellular organismal process | 4 | GO:0032501 |
|  | nucleic acid metabolic process | 4 | GO:0090304 |
|  | cholesterol transport | 1 | GO:0030301 |
| Cellular process | actin cytoskeleton organization | 5 | GO:0030036 |
|  | cell cycle process | 2 | GO:0022402 |
|  | cell recognition | 1 | GO:0008037 |
|  | cellular component organization | 2 | GO:0016043 |
|  | Cellular protein localization | 3 | GO:0034613 |
|  | chromosome organization | 3 | GO:0051276 |
|  | immune response | 2 | GO:0006955 |
|  | immune system process | 3 | GO:0002376 |
|  | Intracellular transport | 4 | GO:0046907 |
|  | ion transport | 1 | GO:0006811 |
|  | protein folding | 9 | GO:0006457 |
|  | response to external stimulus | 2 | GO:0009605 |
|  | vacuole organization | 3 | GO:0007033 |
|  | vesicle-mediated transport | 2 | GO:0016192 |
